# Development of Iron Nanoparticles (FeNPs) Using Biomass of *Enterobacter:* Its Characterization, Antimicrobial, Anti-Alzheimer’s, and Enzyme Inhibition Potential

**DOI:** 10.3390/mi13081259

**Published:** 2022-08-05

**Authors:** Sania Zafar, Shah Faisal, Hasnain Jan, Riaz Ullah, Muhammad Rizwan, Abdullah, Amal Alotaibi, Nadia Bibi, Amin Ur Rashid, Aishma Khattak

**Affiliations:** 1 Institute of Molecular Biology and Biotechnology, Bahauddin Zakariya University, Multan 60000, Pakistan; 2 Department of Life Science, National Tsing Hua University, Hsinchu City 30071, Taiwan; 3 Department of Health and Biological Sciences, Abasyn University, Peshawar 25000, Pakistan; 4 Institute of Biotechnology and Microbiology, Bacha Khan University, Charsadda 24460, KPK, Pakistan; 5 Institute of Biochemical Sciences, National Taiwan University, Taipei 10617, Taiwan; 6 Medicinal Aromatic and Poisonous Plants Research Center, Department of Pharmacognosy, College of Pharmacy, King Saud University, Riyadh 12211, Saudi Arabia; 7 Center for Biotechnology and Microbiology, University of Swat, Odigram, Mingora 19130, Pakistan; 8 Department of Microbiology, Abdul Wali Khan University, Mardan 23200, KPK, Pakistan; 9 Department of Basic Science, College of Medicine, Princess Nourah Bint Abdulrahman University, Riyadh 11671, Saudi Arabia; 10 Department of Microbiology, Shaheed Benazir University, Peshawar 25000, KPK, Pakistan; 11 Department of Applied Physical and Material Sceinces, University of Swat, Odigram, Mingora 19130, Pakistan; 12 Department of Bioinformatics, Shaheed Benazir University, Peshawar 00384, KPK, Pakistan

**Keywords:** FeNPs, *Enterobacter* strain G52, FTIR, anti-diabetic, anti-inflammatory, anti-Alzheimer’s

## Abstract

Nanotechnology is a new field that has gained considerable importance due to its potential uses in the field of biosciences, medicine, engineering, etc. In the present study, bio-inspired metallic iron nanoparticles (FeNPs) were prepared using biomass of *Enterobacter* train G52. The prepared particles were characterized by UV-spectroscopy, TGA, XRD, SEM, EDX, and FTIR techniques. The crystalline nature of the prepared FeNPs was confirmed by XRD. The SEM techniques revealed the particles size to be 23 nm, whereas in FTIR spectra the peaks in the functional group region indicated the involvement of bioactive compounds of selected bacterial strains in the capping of FeNPs. The EDX confirmed the presence of iron in the engineered FeNPs. The FeNPs were then evaluated for its antibacterial, antifungal, antioxidant, anti-inflammatory, anti-Alzheimer’s, anti-larvicidal, protein kinase inhibition, anti-diabetic, and biocompatibility potentials using standard protocols. Substantial activities were observed in almost all biological assays used. The antioxidant, anti-cholinesterase, and anti-diabetic potential of the prepared nanoparticles were high in comparison to other areas of biological potential, indicating that the FeNPs are capable of targeting meditators of oxidative stress leading to diabetes and Alzheimer’s disease. However, the claim made needs some further experimentation to confirm the observed potential in in vivo animal models.

## 1. Introduction

Oxidative stress is encountered in human bodies whenever the production of reactive oxygen species (ROS) is high [1]. Normally, they are promptly detoxified by antioxidant enzymes as the body is equipped with an efficient antioxidant system [2]. Nearly one-fourth of oxygen we inhale is converted into ROS [3]. Despite the heavy burden of the ROS, humans have further worsened the situation by depending more on synthetic/processed foods which further give rise to other free radicals for which the body does not have sufficient antioxidant enzymes to detoxify [4]. This condition leads to a number of health complications, such as: diabetes; neurodegenerative diseases, such as Alzheimer’s; cardiovascular diseases; and even cancer [5]. In Alzheimer’s disease, along with other complications, there is a deficit in acetylcholine for which the cholinesterase inhibitors are used as an antagonist [6]. Oxidative stress is the key factor in aging, diabetes, Alzheimer’s, and dementia, etc. [7]. There is a dire need to scavenge the mediators of stress. Related to this, efforts are being made from almost all research angles to lessen the complications of the mentioned diseases on human [8]. Nanotechnology is the emerging research field, the science of reducing the material particle sizes to nanometer scale range (below 100 nm), that has shown applications in a range of different fields of science, and especially in the medical sciences [9]. A number of approaches are being used in fabricating the nanoparticles, broadly categorized into chemical, biological, and physical approaches [10]. The nanoparticles have also been reported to have other areas of biological potential, such as antimicrobial, anti-inflammatory, larvicidal, etc. [11].

Several methods have been reported for the synthesis of nanoparticles as mentioned above [10]. The same element nanoparticles have different sizes if produced by two or more than two different methods because the particles precipitated quickly have smaller sizes rather than in a method where they are precipitated slowly [12]. The traditional NPs fabrication protocols, such as hydrothermal, sol-gel, and sonochemical, have disadvantages, such as high manufacturing costs, low production rate, and a high energy need [13]. Chemical approaches (e.g., precipitation, hydrothermal, sonochemical, etc.) include the use of noxious chemicals, the generation of perilous side-products, and pollution from noxious substances [14]. As a result, there is a need to develop NPs with greener routes, involving plants/microbes waste biomass as a reductant, that are clean, nontoxic, and ecologically friendly [15]. Some of the major benefits include the fact that they may be created in short time, whereas micro-organism-assisted techniques require longer time due to microbial culture and growth [16].

The biologically inspired methodologies in fabricating the NPs have shown enhanced structural/morphological and biological properties [17]. Several natural materials have been employed as reducing/capping agents in the fabrication of NPs, namely plant/bacterial extracts, and biomaterials [18]. The biologically generated FeNPs have shown diverse applications in different fields, including sensors, chemical reaction catalysts, bio-labeling, photo catalysts, and as antimicrobial agents [19]. Several researchers have used micro-organisms, most notably *Fusarium oxyporum*, as a bio-reductant for the synthesis of FeNPs [20], believing that the growth of micro-organisms is high in comparison to plants [21]. The biomass of *F. oxyporum* and the extracted constituents from this fungus have converted silver ions to stable AgNPs [22]. Moreover, the biomass of bacteria and fungus, or the extracted bio-compounds from these microbes, have also been employed for the synthesis of FeNPs [23]. 

To the best of our knowledge, *Enterobacter* strain G52 biomass has not been used in the fabrication of iron nanoparticles. The aim of the present study was to synthesize FeNPs using *Enterobacter* strain G52 biomass as a reductant. The synthesized FeNPs were characterized by UV-spectroscopy, XRD, SEM, TGA, EDX, and FTIR techniques to visualize their morphology, involvement of bioactive compounds, stability, crystallinity of the particles, etc. Furthermore, antibacterial, antifungal, antioxidant, anti-inflammatory, anti-leishmanial, anti-Alzheimer’s, anti-larvicidal, protein kinase, anti-diabetic, and biocompatibility were also evaluated following standard protocols. 

## 2. Materials and Methods

### 2.1. Biosynthesis of FeNPs Using Enterobacter Strain G52

FeNPs were synthesized using a previously described method, with few new modifications [24]. *Enterobacter* strain G52 was isolated from the hospital and identified (Gene bank accession number JF783991.1). *Enterobacter* strain G52 was inoculated in a nutrient broth (NB) and incubated for 24 h, before being diluted four times with fresh NB to a final volume of 100 mL and incubated for another 24 h. Using the bacterial synthesis method of NPs, *Enterobacter* strain G52 was used to reduce and stabilize Fe^+^ ions. In a 100 mL Erlenmeyer flask, 1:10 reaction mixtures were prepared by mixing 2 mL biomass with 20 mL of 13 mM Fe_2_SO_4_·7H_2_O solution at room temperature. The solutions were stirred constantly at 60–70 °C for 8 h before being stirred again at 37 °C for 24 h. At 14,000 rpm for 10 min, multiple centrifugations were used to wash and purify FeNPs and then dried at 80 °C. The dried powder was collected and stored carefully at 4 °C in airtight vial for all subsequent characterization and biological research.

### 2.2. Characterization of Biosynthesized FeNPs

Advanced tools were used to assess the physicochemical and morphological features of biosynthesized FeNPs [25]. UV-spectroscopy uses the typical range of 200 to 700 nm to monitor the interaction between biomass and metallic salt. The crystal nature of biologically synthesized FeNPs was determined using the X-ray diffraction (XRD) profile. The Panalytical’s X’Pert X-ray diffractometer was utilized to produce the XRD peaks at CuKα (=1.54056 Ǻ). FeNPs were studied using Fourier transform infrared (FTIR) spectroscopy in the 400–4000 cm^−1^ spectrum region to reveal and assess related functional groups involved in their biosynthesis approach [26]. Scanning electron microscopy was applied to measure the physical dimensions and morphological characteristics of biosynthesized FeNPs (JSM-5910, Japan) The biosynthesized FeNPs were subjected to energy dispersive X-ray (EDX) spectroscopy to assure their elemental composition. Thermo galvanometric analysis (TGA, TGA801) was employed to find out the phase formation and thermal stability of FeNPs.

### 2.3. Antibacterial Activity

The agar well diffusion technique was used to evaluate the antimicrobial efficacy of FeNPs [27]. The microbes tested in the study included *P. stuarti*, *P. aeruginosa*, *S. aureus*, and *E. coli*. The McFarland standards were employed to control bacterial cultures to an optical density of (OD = 0.5). After that, cotton swabs were applied to evenly spread 50 µL of fresh culture onto nutrient agar plates. Then, 5 mm wells were made using a sterile borer, and 10 µL of the tested samples were added, along with appropriately labeled plates. The study used kanamycin and DMSO as positive and negative controls, respectively. The culture plates were then incubated for 24 h at 37 °C. After the incubation period, zones of inhibition (ZOI) were measured in millimeters (mm).

### 2.4. Antifungal Activity

FeNPs were tested against four spore-forming fungi, including *Fusarium solani* (FCBP 434), *Aspergillus fumigatus* (FCBP 66), *Aspergillus niger* (ATCC 1015), and *Aspergillus flavus* (ATCC 1015) [28]. Solution (0.02% *v*/*v*) was used to make spore suspension from stock cultures for each fungal strain. First, a 50 µL aliquot of the suspension was swabbed well and put on several Petri plates with sterilized SDA medium. Next, 10 µL FeNPs were then inserted into each well inside the solidified medium under very sterile conditions. DMSO was employed as a negative control and Clotrimazole was employed as a positive control. After 48 h of incubation period, zones of inhibition (ZOI) were measured using a vernier caliper.

### 2.5. Anti-Inflammatory Activities

#### 2.5.1. Against COX-1 and COX-2

The inhibitory potential of COX-1 (Ovine kit 701,050 France) and COX-2 (Human kit 701,050 France) for testing materials was assessed. Ibuprofen (10 M) was employed as a positive control, whereas arachidonic acid (1.1 mM) was utilized as a reagent. Both COXs were recorded according to the equipment’s manufacturer’s specifications. In a 96-well plate, the test was performed in triplicates. A Synergy II reader was applied to measure N, N, N/, N/tetramethyl-p-phenylenediamine at 590 nm in a 96-well microplate.

#### 2.5.2. Against 15-LOX

FeNPs were assessed for their inhibitory potential against 15-LOX (760,700 kit, Cayman, France). Then, 100 μM nordihydroguaiaretic acid (NDGA) and 10 μM arachidonic acid were used as positive and negative controls, respectively. The quantity of hydroperoxides produced by the lipo-oxygenation process in 10 mM Tris-HCl buffer at a 7.4 pH filter supplied in the kit was measured using the soy 15-lipooxygenase standard. The FeNPs and enzyme were loaded in a 96-well plate and incubated for 5 min before recording the absorbance at 940 nm with a Synergy II reader (BioTek Instruments, Colmar, France). After 5 min of incubation, the inhibitor was loaded into the enzyme mixture, and the absorbance was recorded. After 5 min of incubation, the substrate was added to the pre-incubated sample, followed by the chromogen, and the absorbance was recorded.

#### 2.5.3. Against Secretory Phospholipase A2 (sPLA2)

The inhibitory potential of FeNPs was assessed against sPLA2 using an assay kit (10,004,883, Cayman Chem. Co, Montluçon, France). Then, 4 mm diheptanoyl thio-PC and 100 mm thiotheramide-PC acted as standard controls. The diheptanoylthio-PC ester is cleaved, releasing free thiols, which were identified using DTNB at 420 nm in a 96-well microplate. 

### 2.6. Anti-Larvicidal Activity

FeNPs were studied for their anti-larvicidal activity against the larvae of dengue vector, *Aedes aegypti* L. The previously used standard procedure was applied with minimal modifications [29]. Five groups were employed, four of which included 25 third instar larvae each (for varying concentrations), and one of which served as a control group. In a sterile well plate, each well contained 25 third instar larvae in 200 mL of the FeNPs solution at the appropriate concentration and distilled water as a negative control. The well plate was incubated under conventional insectary settings, which included a 12 h light to 12 h dark photoperiod, a temperature of 28 °C, and an 80% relative humidity. The larvae were not fed during the trial. After 24 h, the percentage of those who died was analyzed. Those specimens that could not move or moved sluggishly in response to tactile stimulation were counted as dead. The experiment was repeated five times, with the percentage mortality representing the average of the five triplicates.

### 2.7. Anti-Leishmanial Avtivity

The leishmanicidal potential of FeNPs against both promastigote and amastigote were investigated using a well-established method [30]. The *Leishmania* KWH23 strain was grown and incubated in MI99 media supplemented with 10% FBS. Then, 20 µL of FeNPs and 180 µL of culture solution were placed in each well of a 96-well plate. At 25 °C, the samples were incubated for 72 h. Amphotericin-B and DMSO 1% in PBS were used as controls, respectively. After incubation, each well was filled with 20 µL of MTT solution (4 mg/mL in distilled H_2_O), and the culture plate was re-incubated at 25 °C for another 4 h. A 96-well microplate reader was applied to record the absorbance at 540 nm. The following formula was employed to compute the percentage inhibition:

$$\%\;\mathrm{Inhibition}=\left[1-\left\{\frac{\mathrm{Absorbance}\;\mathrm{of}\;\mathrm{sample}}{\mathrm{Absorbance}\;\mathrm{of}\;\mathrm{control}}\right\}\right]\times 100$$

### 2.8. Anti-Alzheimer’s Activity

The capacity of FeNPs to inhibit acetylcholinesterase (AChE) and butyrylcholinesterase (BchE) were checked at different concentrations, ranging from 62 to 1000 µg/mL [31]. Phosphate Buffer Saline 5, 5-dithiobisnitrobenzoic acid, acetylcholine iodide, and butyrylcholine iodide were used to make a substrate solution. The pristine reaction mixture was employed as a positive control, while galanthamine hydrobromide (5 mg/0.5 mL methanol) was employed as a negative control. Finally, the absorbance of the test samples at 412 nm was determined. 

### 2.9. Protein Kinase Inhibition Activity

The protein kinase inhibition potentials of bioinspired FeNPs were evaluated with minimum adjustments to a technique developed by [32]. In this work, the *Streptomyces* 85E strain was cultured using sterile ISP4 media. Thin wells were produced in media, 100 µL of FeNPs were added to each well. Surfactin was applied as a positive control, whereas DMSO was employed as a negative control. The plates were then incubated at 28 °C for 2 days. After two days, clean and bald zones around wells were measured.

### 2.10. Anti-Diabetic Activity

The anti-diabetic potential of FeNPs were assessed employing α-amylase and α-glucosidase inhibition tests. 

#### 2.10.1. α-Amylase Inhibition

The potential of FeNPs was assessed using an α-amylase test via a well-established technique with just minor changes [33]. A 96-well microplate was used for this experiment. Each well in the test was filled with α-amylase (25 µL), phosphate buffer (15 µL), starch (40 µL), and FeNPs sample (10 µL), and then incubated for 30 min at 50 °C. Next, 20 µL of 1 M HCl and 90 µL of iodine solution were loaded to each well. In this experiment, Acarbose was employed as a positive control, and DMSO was used as a negative control. The absorbance of the test samples was recorded at 540 nm. Using the following formula, the inhibition was determined as a percentage.

$$\%\;\mathrm{Enzyme}\;\mathrm{inhibition}=\frac{\left(\mathrm{Abs}\;\mathrm{sample}-\mathrm{Abs}\;\mathrm{negative}\;\mathrm{control}\right)\times 100}{\left(\mathrm{Abs}\;\mathrm{blank}-\mathrm{Abs}\;\mathrm{negative}\;\mathrm{control}\right)}$$

#### 2.10.2. α-Glucosidase Inhibition

The α-glucosidase inhibition potentials of FeNPs were evaluated using a modified protocol [33]. To dissolve α-glucosidase, 50 mL of phosphate buffer (pH 6.8) and 100 mg of bovine serum albumin was poured simultaneously. After 5 min, 490 μL of phosphate buffer (pH 6.8) and 250 μL of p-nitrophenyl-D-glucopyranoside (5 mM) were mixed and then was kept at 37 °C. After that, FeNPs were treated with 250 μL α-glucosidase for 15 min at 37 °C. After stopping the reaction with a 2 mL Na_2_CO_3_ (200 mM) solution, absorptions were measured at 400 nm. The experiment was repeated three times and, in this experiment, the acarbose was used as a positive control.

$$\%\;\mathrm{Enzyme}\;\mathrm{inhibition}=\frac{\left(\mathrm{Abs}\;\mathrm{sample}-\mathrm{Abs}\;\mathrm{negative}\;\mathrm{control}\right)\times 100}{\left(\mathrm{Abs}\;\mathrm{blank}-\mathrm{Abs}\;\mathrm{negative}\;\mathrm{control}\right)}$$

### 2.11. In Vitro Hemolysis Activity

Fresh human red blood cells (hRBCs) were employed to test the biocompatibility study of FeNPs [34]. Healthy volunteers gave their consent before having 1 mL of blood drawn and placed in an EDTA tube. To separate RBCs, blood samples were centrifuged for 7 min at 12,000 rpm. The cells were recovered after three PBS washes, and the supernatant was discarded. To generate a PBS-erythrocyte suspension, 200 μL of erythrocytes were combined with 9.8 μL of PBS (pH: 7.2). An erythrocyte suspension and various concentrations of biologically fabricated FeNPs were mixed and incubated at 35 °C for 1 hr in Eppendorf tubes. After centrifuging the mixture at 1000 rpm for 5 min, the supernatant was loaded onto a 96-well plate, and hemoglobin-releasing absorption spectra at 540 nm were recorded. A positive control of 0.5 percent Triton X-100 was employed, and DMSO was employed as a negative control. The fraction of hemolysis was determined applying the formula below:

$$\%\;\mathrm{Hemolysis}=\frac{\left(\mathrm{Abs}\;\mathrm{sample}-\mathrm{Abs}\;\mathrm{negative}\right)\times 100}{\left(\mathrm{Abs}\;\mathrm{positive}-\mathrm{Abs}\;\mathrm{negative}\right)}$$

### 2.12. Antioxidant Activity

#### 2.12.1. Total Antioxidant Capacity Determination (TAC)

The sample’s total antioxidant capacity was determined using tests developed by Ref. [35]. Then, 100 µL of material was pipetted into the Eppendorf tubes in the experiment using a micropipette. The TAC reagent was then transferred to the Eppendorf tubes holding the examined samples (0.6 M sulphuric acid, 28 mM sodium phosphate, and 4 mM ammonium molybdate in 50 mL dH_2_O). The reaction mixture was incubated for 2 h in a water bath at 90 °C, following which the samples’ absorbance was measured in a microplate reader at 630 nm. TAC was computed 3 times as an ascorbic acid equivalent/mg of the sample.

#### 2.12.2. Total Reducing Power Determination (TRP)

To assess the sample’s overall reduction power, the procedure outlined by [36] was performed three times. The test sample 100 µL, 400 µL of 0.2 Molar phosphate buffer (pH 6.6), and potassium ferric cyanide (1 percent *w*/*v*) were placed in Eppendorf tubes and incubated in a water bath at 55 °C for 30 min. After that, each Eppendorf tube was filled with 400 µL of 10% *w*/*v* trichloroacetic acid and centrifuged at 3000 rpm for 10 min. The supernatant (140 µL) from each combination was put into matched wells of a 96-well plate that had previously been filled with 60 µL of ferric cyanide solution (0.1% *w*/*v*). A microplate reader was used to measure the absorbance of the samples at 630 nm. The approach for both positive and negative controls was the same as for the positive controls. 

#### 2.12.3. Free Radical Scavenging Assay (FRSA)

The procedure initially described by [37] was adopted with minor changes. To examine whether FeNPs might scavenge free radicals, they were evaluated using the DPPH reagent at doses ranging from 12.5 µL to 400 µL. A 96-well plate was filled with 10 µL of testing materials in each well. After that, the DPPH reagent (190 µL) was applied to each sample well. It was then incubated in the dark for 60 min at 37 °C. A positive control, ascorbic acid, was employed, whereas a negative control, DMSO, was used. A microplate photometer was also used to assess the absorbance rate at 515 nm. The FRS potential of biosynthesized FeNPs was calculated as %.

$$\left(\%\right)\;\mathrm{FRSA}\;=\left(1-\frac{Abs}{Abc}\;\right)\times 100$$

The absorbance of the negative control and sample, respectively, is Abc and Abs.

#### 2.12.4. Trolox Antioxidant Assay (ABTS)

A modified ABTS test was used to determine the antioxidant capability of the biosynthesized NPs [38]. In equal portions, potassium per sulphate (2.45 mM) and 7 mM ABTS salt were mixed and incubated at room temperature overnight. After incubation, samples were put in the mixture and kept at room temperature in the dark for 15 min. The BioTek ELX800 was used to measure the absorbance of the sample in the reaction mixture at a wavelength of 734 nm. The Trolox reagent was utilized as a positive control in this test, whereas DMSO was employed as a negative control. In a triplet experiment, the findings (antioxidant potential) were represented as TEAC.

## 3. Results

### 3.1. FeNPs Synthesis

**Figure 1:**
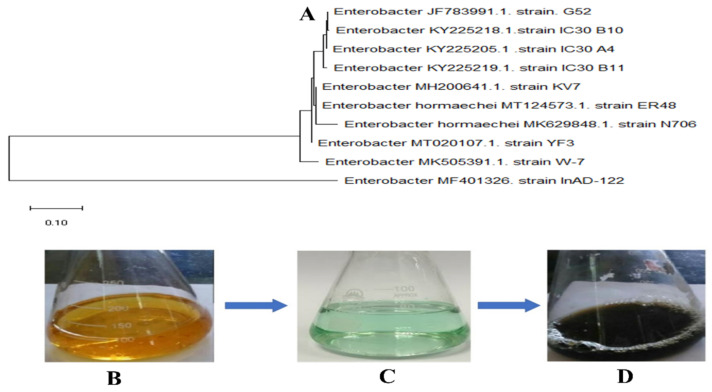
(**A**) Schematic study of the *Enterobacter* strain G52 16S rDNA sequence employed in the production of FeNPs; (**B**) the entire process of making FeNPs is depicted in this schematic picture, *Enterobacter* strain G52 filtrate; (**C**) Fe_2_SO_4_·7H_2_O solution (13 mM); and (**D**) reduction and capping of Fe ions by *Enterobacter* strain G52 filtrate after 24 h.

The bacteria were identified as *Enterobacter* strain G52 based on homology and alignments with previously known bacterial rDNA genomes (Figure 1A), and the data were reported to the NCBI Genebank database. Metallic nanoparticles are known to be produced by various microbes, because the bacterial metabolites control the morphology, size, and properties of the NPs, they have qualities such as chemically produced materials. The micro-organisms used in the production process have a significant impact on the NPs’ physical qualities, such as size, shape, and crystallinity. The formation of a black precipitate confirmed the fabrication of FeNPs using bacterial biomass and metal salt. Due to the reduction in iron ions, a black precipitate developed when the 13 mM Fe_2_SO_4_·7H_2_O solution was added to the bacterial culture drop by drop, and the reaction mixture color altered from colorless to black confirming the production of FeNPs, as shown in Figure 1B–D. After 24 h, a black precipitate formed, indicating that iron ions had been reduced and FeNPs had formed. Once the reaction was completed the mixture was placed in falcon tubes and centrifuged at 10,000 rpm to remove extra salt residues and bacterial biomass. Subsequently, the NPs were washed 3 times with distilled water and dried in an oven at 80 °C. The dried NPs were then calcinated at 500 °C for 2 h to remove suspended salt and biomolecules traces and stored in airtight glass vials at 4 °C for further analysis and biological applications. 

### 3.2. UV-Spectroscopic Analysis

**Figure 2:**
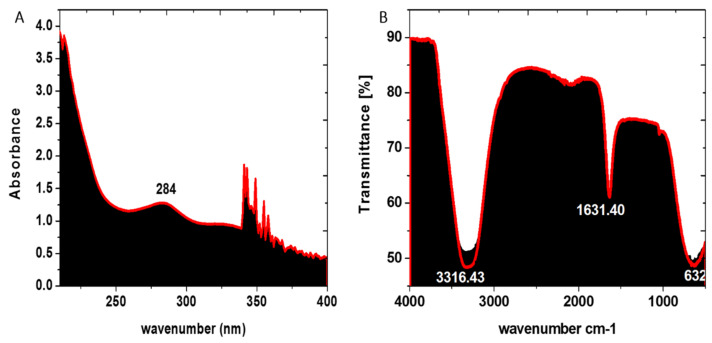
(**A**) UV-Vis spectroscopy of *Enterobacter* strain G52 extract mediated FeNPs and (**B**) FTIR spectra of *Enterobacter* strain G52 extract mediated FeNPs.

The initial color change in the reaction mixture shows that biomolecules produced by *Enterobacter* strain G52 have reduced Fe^+^ ions to Fe^0^. Following the color change allows for a proper reduction before being tested for absorbance using a spectrometer at a certain wavelength (Shimadzu UV-1800). FeNPs absorbed a portion of the characteristic wavelength, resulting in a Surface Plasmon Resonance peak at 284 nm in Figure 2A. FeNPs were effectively synthesized, as evidenced by the distinctive absorbance peak. Similarly, FeNPs were synthesized using bacterial biomass for the UV investigation, and the UV spectrum revealed an absorption band at 290 nm.

### 3.3. FTIR Analysis

The existence of diverse functional groups accountable for the production of FeNPs in bacterial biomass was explored using FTIR spectroscopy. The vibrational bands of FeNPs in the wavelength range between 1000 and 3500 cm^−1^ are depicted in Figure 2B. Characteristic peaks were found at 1631.40 and 3316.43 cm^−1^. The vibrational band at 3316.43 cm^−1^ indicated O-H stretching due to the involvement of phenol or alcohol as a functional group, whereas the stretching band at 1631.40 cm^−1^ represented C=C stretching due to the olefinic functional group. The existence of iron-oxygen (Fe-O) proved that the produced NPs are FeNPs, and proved the formation of well-defined peaks at 632 cm^−1^. The appearance of functional group signals in the bacterial biomass suggested that certain molecules were bonded to the surface and stayed despite repeated washing. Similar reports were induced by Smith and his colleagues using bacteria to carry out the green synthesis of FeNPs. 

### 3.4. Thermal Properties

**Figure 3:**
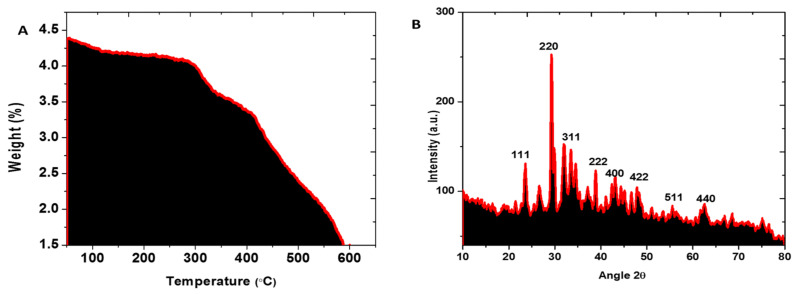
(**A**) TGA micrograph of *Enterobacter* strain G52 extract mediated FeNPs and (**B**) X-ray diffraction pattern of FeNPs.

As shown in Figure 3A, the thermal nature of synthesized FeNPs was investigated using TGA assessment in the temperature range of 25 °C to 600 °C. FeNPs lost 30% of their weight till they reached 600 °C. The loss of moisture content from the synthesized samples are responsible for the first weight loss up to 150 °C [12]. As a result of our findings, biosynthesized FeNPs have a higher thermal conductivity.

### 3.5. XRD Analysis of FeNPs

The XRD profile was used to evaluate the crystal structure of the produced FeNPs. Figure 3B displays the XRD data of synthesized FeNPs. The XRD profile shown in this study is very consistent with the JCPDS file 019–0629, with distinctive peaks at 2θ of 20.75°, 31.71°, 36.59°, 38.98°, 45.45°, 53.49°, 56.44°, and 61.11°, corresponding to reflections of the face centered cubic phase of (111), (220), (311), (222), (400), (422), (511), and (440) lattice planes, respectively. The average crystal size of the biosynthesized FeNPs at (111) was determined applying the Debye–Scherrer equation, and was found to be 20.7 nm.

### 3.6. EDX and SEM Analysis

**Figure 4:**
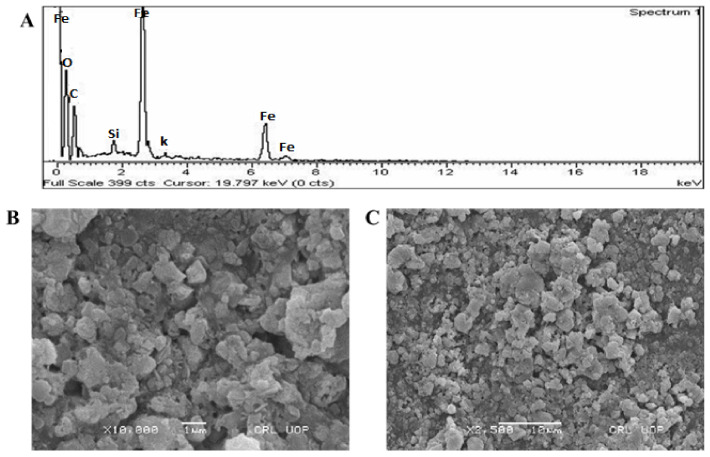
(**A**) Typical EDX spectrum; (**B**) SEM micrographs at 1 µm; and (**C**) SEM micrographs at 10 µm.

As shown in Figure 4A, an intense EDX spectrum for iron was identified, indicating the production of FeNPs. The EDX spectrum has extra peaks, such as chlorine, carbon, oxygen, potassium, and silicon. The bioactive compounds in *Enterobacter* strain G52 encircled the Fe ions during NPs production and formed the extra peaks. SEM was used to assess the overall look of FeNPs in this study. The SEM micrograph of the FeNPs presented in Figure 4B confirms their hexagonal and rectangular morphology. The presence of hexagonal and rectangular shapes of FeNPs with a mean diameter of 23 nm was seen in SEM images at various magnifications, demonstrating their existence.

### 3.7. Antibacterial Assay

In this work, the bactericidal efficacy of FeNPs was examined against four pathogenic bacteria. Several doses were utilized to evaluate bacterial susceptibility, ranging from 25 µg/well to 100 µg/well. All the strains examined were sensitive to FeNPs, with *Pseudomonas aeruginosa* and *Providencia stuartii* being the most susceptible, with zones of inhibition (ZOI) of 15.51 ± 0.27 mm and 18.19 ± 0.64 mm, respectively, as shown in Table 1. Antimicrobial resistance (AMR) and antibiotics have now become unavoidable, and make it necessary to discover new therapeutic tactics for developing effective antibiotics. From the results, all the examined bacterial strains were sensitive to FeNPs in a dose-dependent manner.

### 3.8. Antifungal Assay

For antifungal assessment, the synthesized FeNPs were tested against four fungal strains. Table 2 displays the antifungal presentation of the synthesized FeNPs. At 200 µg/well, *A. nigr, F. solani*, and *A. flavus* demonstrated the highest ZOI with measurements of 12.2 ± 0.1 mm, 15.4 ± 0.3 mm, and 13.5 ± 0.13 mm, respectively. At the same time, the lowest zone of inhibition for *A. Fumigatus* was 10.3 ± 2.13 mm at 25 µg/well. According to studies, the interaction of ROS with fungal hyphae and spores inhibits fungal development. FeNPs might be employed as a potent antimicrobial agent in pristine form or as a carrier for antibiotics and antifungals due to their high antibacterial and antifungal activity.

### 3.9. Anti-Inflammatory Assay

**Figure 5:**
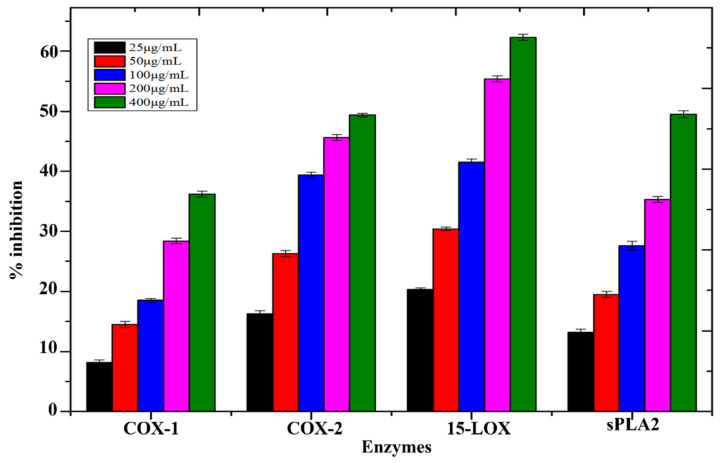
Anti-inflammatory potential of biosynthesized FeNPs.

Tested NPs showed moderate inhibition potential against 15-LOX (62.3 ± 0.5), sPLA2 (49.5 ± 0.6), and COX-2 (49.6 ± 0.3), followed by COX-1 (36.2 ± 0.4), respectively. Figure 5 depicts the percent inhibition of the other tested samples. FeNPs have been demonstrated to have improved anti-inflammatory efficacy in previous investigations. FeNPs are responsible for suppressing enzymes that induce inflammation in the body.

### 3.10. Anti-Larvicidal Assay

Material scientists have recently been increasingly focused on finding superior alternatives in plant extracts and bacteria-assisted NPs against the proposed vector for dengue virus spread. In our work we have also made a possible approach to evaluate our synthesized NPs against *Aedes aegypti*. A variety of concentrations of synthesized NPs (25, 50, 100, 150, and 200 ppm) were evaluated against *Aedes aegypti* second and fourth instars in this assay. At 200 ppm, 45.2 ± 0.3 percent mortality was found, followed by 12.6 ± 0.4 percent at 25 ppm. Mortality decreases in lockstep with lower doses, as shown in Table 3.

### 3.11. Anti-Leishmanial Assay

We used the MTT test to evaluate the anti-leishmanial efficacy of biogenic FeNPs at various concentrations, ranging from 50 to 400 µg/mL against amastigotes and promastigotes, as shown in Table 4. The biogenic NPs had a potent death rate of 62.4 ± 1.19 for promastigotes and 57.5 ± 1.09 for amastigotes at the maximum 400 µg/mL concentration. For promastigote, the lowest mortality rate was 20.77 ± 0.79, while for amastigote, it was 17.72 ± 0.41 at 50 µg/mL. Our findings are consistent with those of prior research.

### 3.12. In Vitro Anti-Alzheimer’s Assay

This study investigated the inhibitory response of two cholinesterase enzymes, AChE and BChE, at doses ranging from 62.5 µg/mL to 1000 µg/mL of FeNPs, as shown in Table 5. The inhibitory responses of both the enzymes were revealed to be dose-dependent. At 1000 µg/mL, the NPs were extremely active, inhibiting AChE by 43.41 ± 0.32% and BChE by 57.57 ± 0.63%. At 62.5 µg/mL, AChE had a 12.57 ± 0.92% inhibition response, whereas BChE had an 18.24 ± 0.13% inhibition response. As a result, FeNPs have the potential to be exploited for the targeted administration of prospective Alzheimer’s treatments. However, more research is needed to improve biocompatibility and study FeNPs in vivo toxicity.

### 3.13. Protein Kinase Inhibition Assay

The capacity of biogenic FeNPs to inhibit protein kinases was revealed utilizing the *Streptomyces* 85E strain. Clear bald zones were found for each concentration of FeNPs assessed, with the largest bald zone measuring 12.52 ± 0.31 at 5 mg/mL and the minor bald zone measuring 2.24 ± 0.28 at 500 µg/mL. Overall, the findings revealed that biogenic NPs obtain substantial capping and stabilizing elements from bacterial biomass, accountable for their anti-cancerous properties. As shown in Table 6, *Streptomyces* strains were observed to be inhibited by biogenic FeNPs in a dose-dependent approach.

### 3.14. Anti-Diabetic Assay

As shown in Table 7, FeNPs samples were assessed for inhibition of α-amylase and α-glucosidase. The inhibitory efficacy of α-amylase and α-glucosidase was observed to be relatively high. At 400 µg/mL, maximal inhibition for α-amylase was revealed to be 51.32 ± 0.39 and 58.37 ± 0.68 for α-glucosidase, respectively. We showed here that bio-based NPs can have strong anti-diabetic properties and can be used as an efficacious therapeutic drug for the therapy of diabetes as an effective solution to expensive and ineffective medicines. 

### 3.15. In Vitro Hemolysis Activity

The biocompatibility of the green synthesized FeNPs were proven using hRBCs in a biocompatibility experiment. The hemolysis of hRBCs against varied doses of FeNPs (50 µg/mL to 400 µg/mL) is seen in this study. Table 8 presents the biocompatibility outcomes of our experiment. According to the American Society for Testing Materials’ biocompatibility recommendations, substances with hemolysis of less than 2% are deemed non-hemolytic, those with hemolysis of 2–5% are considered somewhat hemolytic, and those with hemolysis of more than 5% are classified hemolytic. There are several aspects to examine, as shown in Table 1, all of our stock solutions of manufactured NPs evince minimal hemolysis, displaying their good biocompatibility even at maximum concentrations. To employ NPs in biomedical applications, we must first determine their biocompatibility. Even at maximum concentrations of 400 µg/mL, as-synthesized FeNPs are hemocompatible; no hemolytic efficacy is seen at this dose. From the study’s outcomes, as-synthesized FeNPs are safe, and they might be employed for therapeutic purposes.

### 3.16. Antioxidant Assay

**Figure 6:**
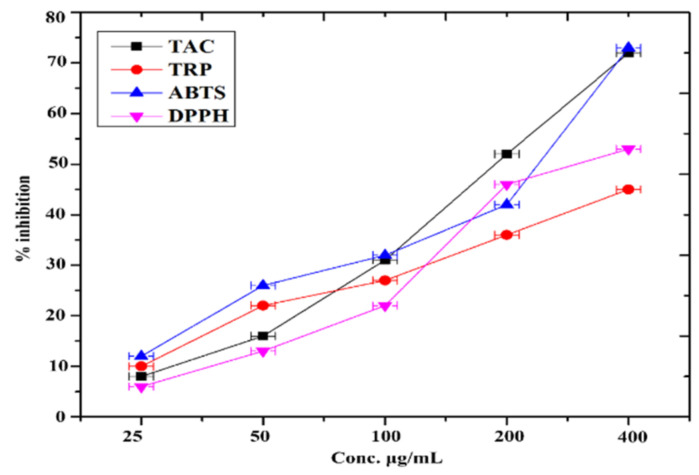
Antioxidant potential of synthesized of FeNPs.

The antioxidant potential of FeNPs was assessed using the total antioxidant capacity (TAC), total reducing power (TRP), ABTS, and DPPH-free radical scavenging assays (FRSA). Figure 6 summarizes the findings. In all of the assays, FeNPs displayed exceptional dose-dependent antioxidant activity. The greatest antioxidant capacity in terms of ascorbic acid equivalents was determined to be 84.35 ± 0.86 μgAAE/mg at a concentration of 400 μg/mL. The maximum TAC and ABTS free radical scavenging activity was 72.24 ± 0.75% and 73.12 ± 0.83% TEAC, respectively, at 400 µg/mL.

## 4. Discussion

Because of its simplicity of use and cost efficiency, as well as its potential for large-scale manufacturing, the NPs synthesis approach has piqued the scientific community’s attention in recent years. We used a biosynthesis approach to make FeNPs from *Enterobacter* strain G52, and we tested the NPs against urinary tract infection (UTI) isolates. Furthermore, there is no evidence in the literature to support the anti-Alzheimer’s effects of bacterial biomass produced FeNPs. In the first steps, the FeNPs were explored for their morphological features employing diverse analytical tools, including UV-spectroscopy, FTIR, TGA, XRD, SEM, and EDX. According to UV-spectroscopy, the sample absorbed energy at 284 nm, which is a sign for typical peak value for FeNPs. The results were validated by X-ray spectroscopy [25]. Aside from that, an absorption peak at 284 nm with no other peak demonstrated the NPs exceptional purity. Many investigations revealed a significant absorption peak of FeNPs below 374 nm wavelengths, related to the sample red shift at 500 °C and 700 °C [39]. 

The vibrations of alkanes, phenol, alcohols, aromatics, alkenes, alkyl-halides, and aliphatic amines were revealed by FTIR analysis of iron nanoparticles which are in accordance with previous results [40]. Furthermore, –C=O–, C–O–C, and C–O stretching vibrations were shown to generate maxima in carboxylic acid, polysaccharide, and amino acid, respectively [41]. The produced FeNPs has crystalline sizes in the range of 23 nm, as estimated by Nano-measurer and ImageJ analysis, as confirmed by SEM micrographs. The size of NPs was larger in this work than in [42], which might be attributed to changes in synthesis settings, such as temperature, incubation period, bacterial extract type, and handling applications. 

Furthermore, an EDX analysis showed pure FeNPs phases and a strong peak in the EDX spectrum, showing that the test sample contained pure iron. The EDX spectra of FeNPs were obtained using a simple precipitation process using iron as the starting material. Pure FeNPs with substantial peaks have been successfully synthesized, according to the EDX spectrum. However, additional peaks in the spectrum were detected, suggesting that bacterial biomolecules were involved in nanoparticle synthesis. Throughout, in correlation with other reports, we found the same EDX pattern of FeNPs with great purity [43]. The XRD profile was employed to assess the size and crystallinity of the bio-fabricated FeNPs. The XRD spectrum demonstrated the planar alignment and crystalline structure of FeNPs as 56.44, 53.49, 45.45, 38.98, 36.59, 31.71, and 20.75 degrees at 2Theta. Numerous XRD reflection planes indicate that the centered cubic structure, as attested by JCPDS Card No. 36–1451. According to Scherer’s equation, the average crystal size is 20.7 nm [44].

After thorough morphological and chemical analysis, the produced NPs were tested for a range of biological applications against human pathogenic microbes and several enzymes which are involved in the prognosis of several fatal diseases. UTIs are one of the most prevalent bacterial illnesses, affecting around 150 million individuals worldwide each year. Even though both men and women are susceptible to UTIs, women are more likely to become infected, with up to half of all women being ill at some time in their life. Due to the increasing prevalence of multidrug-resistant (MDR), which render antibiotic therapy for acute infection ineffective [45], current therapeutic options are inadequate. In our experiments, FeNPs showed remarkable bactericidal action against UTI isolates, such as *P. stuarti, P. aeruginosa, S. aureus*, and *E. coli*. As a result of our findings, we may assume that biosynthesized FeNPs have significant bactericidal action in their natural state and that coating pharmaceuticals can boost their effectiveness against MDR bacteria. Our results are incongruent with earlier reports [34]. The antifungal efficacy of bacterial biomass mediated NPs was also studied, with the maximum zones of inhibition (ZOI) recorded against *A. niger, F. solani,* and *A. flavus* (12 ± 2.1 mm, 15.4 ± 0.3 mm, and 13.5 ± 0.13 mm), respectively.

We also checked the inhibition of COX-1, COX-2, sPLA2, and 15-LOX by in vitro to confirm the anti-inflammatory action of the FeNPs. Tested NPs showed moderate inhibition potential against sPLA2 (49.5 ± 0.6), 15-LOX (62.3 ± 0.5); COX-1 (36.2 ± 0.4) and COX-2 (49.6 ± 0.3) had the best anti-inflammatory efficacy among all samples. Similar results were reported by [41]. A variety of concentrations of synthesized NPs (25, 50, 100, 150, and 200 ppm) were evaluated against *Aedes aegypti* second and fourth instars in this work. At 200 ppm and 25 ppm percent mortality of 45.2 ± 0.3% and 12.6 ± 0.4% were found, respectively, in accordance with previous results [46].

Leishmaniasis is a severe worldwide health burden, according to the World Health Organization (WHO), with a wide range of clinical symptoms and possibly deadly consequences. According to the study, 1.5 to 2 million cases occur yearly, placing 350 million individuals at risk [47]. We have tested our synthesized products against both forms of leishmania parasite to provide a framework for proper NPs based anti-leishmanial remedies. At 400 μg/mL, the NPs exposed to promastigote and amastigote parasite types had significant death rates of 62.4 ± 1.19% and 57.5 ± 1.09%, respectively. In accordance with these results, we can believe that FeNPs might be used as a future treatment for cutaneous leishmaniasis. Cholinesterase inhibitors are one of the most effective Alzheimer’s disease (AD) treatments now available, and they may be taken at any stage of the disease. Several synthesized and natural substances were shown to inhibit cholinesterase enzymes efficiently [44]. The FeNPs were plenty efficient at 1000 μg/mL against AChE (43.41 ± 0.32) and BChE (57.57 ± 0.63).

Protein kinase are enzymes that aid cell interaction and progression through the cell cycle by allowing cells to communicate across the nuclear membrane. Tyrosine and protein kinases phosphorylate serine-threonine residues, which is significant in cancer therapy. These residues function in metabolism, cell apoptosis, and cellular proliferation and differentiation regulation and control. Out of control phosphorylation may induce and encourage genomic alterations that lead to cancer [48]. A dose-dependent inhibitory effect for NPs formulations was discovered. At 5 mg/mL, the largest ZOI was found to be 12.52 ± 0.31, while the lowest ZOI was found to be 2.24 ± 0.28 at 0.5 mg/mL. A similar approach was shown in a previous study [49]. Diabetes Mellitus (DM) is a term applied to explain a group of metabolic illnesses characterized by persistent hyperglycemia [50]. When insulin comes into contact with body cells, its synthesis is minimal or inactive, leading to a malfunction. One of the essential tactics for treating diabetes is to reduce postprandial hyperglycemia, which may be performed by blocking the two most essential carbohydrate hydrolyzing enzymes in the digestive tract, α-amylase and α-glucosidase [51]. The effective inhibition of α-amylase and α-glucosidase was investigated at diverse concentration of FeNPs ranging from 400 μg/mL to 25 μg/mL. The inhibitory efficacy of α-amylase and α-glucosidase was observed to be relatively high. At 400 μg/mL, maximum α-amylase inhibition was determined to be 51.32 ± 0.39 and 58.37 ± 0.68 for α-glucosidase, respectively, as previously reported by [52].

Biocompatibility is one of the most crucial aspects of the therapeutic usability of nano-systems. The degree of biocompatibility is determined by physico-chemical properties and the environment to which NPs are exposed [53]. We tested the FeNPs in vitro hemocompatibility because of this importance. Even at the maximal concentration of 400 μg/mL, the NPs exhibited excellent biocompatibility against collected hRBCs. Our findings show that FeNPs generated by *Enterobacter* strain G52 are very biocompatible and may be employed in diverse biological applications. Our results revealed that FeNPs mediated by the *Enterobacter* strain G52 are stable in vitro and might be used for practical medicinal applications.

## 5. Conclusions

In this study, *Enterobacter* bacterial strain G52 biomass-based FeNPs were prepared, characterized by various instrumental techniques, and investigated as therapeutic agents in in vitro for various health complications, especially for those related to oxidative stress. The biomass of *Enterobacter* bacterial strain G52 contains proteins, carbohydrates, and lipids chemicals which helped in the capping and reduction of iron into FeNPs. The prepared nanoparticles showed potent antibacterial and antifungal potential against selected microbial strains. Similarly, the amastigote and promastigote forms of the parasite *Leishmania tropica* were found to be susceptible to the prepared nanoparticles. DPPH, ABTS, α-amylase, α-glucosidase, acetylcholinesterase, and butyrylcholine esterase were potently inhibited by these nanoparticles, indicating that they could be effectively used as therapeutic agents for the oxidative stress and related complications, such as diabetes and Alzheimer’s. The observed therapeutic potentials of the prepared nanoparticles are the in vitro experiment results which need to be further confirmed in in vivo animal models.

## Figures and Tables

**Table 1:** Antibacterial potential of FeNPs against selected bacterial strains.

Strains	FeNPs Conc.				
	25 µg	50 µg	75 µg	100 µg	Control
***Pseudomonas aeruginosa***	11.73 ± 0.27	13.63 ± 0.39	15.49 ± 0.43	15.51 ± 0.27	21.31 ± 0.88
***Providencia stuartii***	12.60 ± 0.47	15.41 ± 0.39	16.53 ± 0.66	18.19 ± 0.64	21.71 ± 1.19
***Escherichia coli***	6.91 ± 0.55	10.63 ± 0.49	12.84 ± 0.73	15.32 ± 0.79	17.47 ± 0.92
***Staphylococcus aureus***	10.3 ± 0.58	12.5 ± 0.40	13.4 ± 0.61	16.7 ± 0.41	18.3 ± 1.04

**Table 2:** Antifungal potential of FeNPs against selected fungal strains.

Strains	FeNPs Conc.				
	25 µg	50 µg	100 µg	200 µg	Control
***Aspergillus niger***	4.6 ± 0.27	6.5 ± 0.39	9.3 ± 0.43	12.2 ± 0.27	14.3 ± 0.88
***Aspergillus flavus***	7.3 ± 0.44	9.4 ± 0.68	11.5 ± 0.61	13.5 ± 0.64	15 ± 0.60
***Fusarium solani***	8.4 ± 0.55	10.3 ± 0.49	12.5 ± 0.73	15.4 ± 0.77	13.4 ± 0.61
***Aspergillus fumigatus***	4.2 ± 0.23	6.4 ± 0.41	8.5 ± 0.49	10.3 ± 0.77	13.1 ± 0.63

**Table 3:** Anti-larvicidal potential of FeNPs against *Aedes aegypti*.

Concentrations	% Mortality
**25 ppm**	12.6 ± 0.4
**50 ppm**	17.4 ± 0.3
**100 ppm**	24.6 ± 0.6
**150 ppm**	32.5 ± 0.4
**200 ppm**	45.2 ± 0.3

**Table 4:** Anti-leishmanial potential of FeNPs against amastigote and promastigote.

Concentration µg/mL	Amastigote	Promastigote
**50**	17.72 ± 0.41	20.77 ± 0.79
**100**	18 ± 0.49	22 ± 0.63
**200**	33 ± 0.44	37 ± 0.88
**400**	57.5 ± 1.09	62.4 ± 1.19

**Table 5:** In vitro cholinesterase potential of FeNPs against AChE and BChE enzyme.

Concentration µg/mL	AChE	BChE
**62.5**	12.57 ± 0.92	18.24 ± 0.13
**125**	21.44 ± 0.99	28.19 ± 0.43
**250**	29.61 ± 0.84	37.44 ± 0.81
**500**	35.27 ± 0.73	47.51 ± 0.69
**1000**	43.41 ± 0.32	57.57 ± 0.63

**Table 6:** Protein kinase inhibition potential of FeNPs.

Concentrations mg/mL	ZOI in (mm)	Control
**0.5**	2.24 ± 0.28	5.53 ± 0.49
**1**	4.93 ± 0.36	8.13 ± 0.64
**2**	8.61 ± 0.36	13.44 ± 0.60
**4**	11.41 ± 0.47	15.68 ± 0.61
**5**	12.52 ± 0.31	17.43 ± 1.13

**Table 7:** Anti-diabetic efficacy of synthesized of FeNPs.

Concentrations µg/mL	α-Amylase	α-Glucosidase
**25**	6.63 ± 0.26	16.53 ± 0.41
**50**	14.88 ± 0.24	23.44 ± 0.69
**100**	27.49 ± 0.31	33.21 ± 0.44
**200**	32.81 ± 0.33	45.72 ± 0.77
**400**	51.32 ± 0.39	58.37 ± 0.68

**Table 8:** Percentage hemolysis of FeNPs.

S.NO	Conc: µg/mL	% Hemolysis
1	400	3.94 ± 0.15
2	200	2.63 ± 0.44
3	100	0.59 ± 0.27
4	50	0.17 ± 0.38

## Data Availability

All required data are present in this file.
